# Population Genetic Structure in Glyphosate-Resistant and -Susceptible Palmer Amaranth (*Amaranthus palmeri*) Populations Using Genotyping-by-sequencing (GBS)

**DOI:** 10.3389/fpls.2018.00029

**Published:** 2018-01-25

**Authors:** Anita Küpper, Harish K. Manmathan, Darci Giacomini, Eric L. Patterson, William B. McCloskey, Todd A. Gaines

**Affiliations:** ^1^Department of Bioagricultural Sciences and Pest Management, Colorado State University, Fort Collins, CO, United States; ^2^Department of Soil and Crop Sciences, Colorado State University, Fort Collins, CO, United States; ^3^Department of Crop Sciences, University of Illinois at Urbana–Champaign, Urbana, IL, United States; ^4^School of Plant Sciences, University of Arizona, Tucson, AZ, United States

**Keywords:** Palmer amaranth, population genetics, glyphosate, herbicide resistance, genetic relatedness, SNP molecular markers, phylogeography

## Abstract

Palmer amaranth (*Amaranthus palmeri*) is a major weed in United States cotton and soybean production systems. Originally native to the Southwest, the species has spread throughout the country. In 2004 a population of *A. palmeri* was identified with resistance to glyphosate, a herbicide heavily relied on in modern no-tillage and transgenic glyphosate-resistant (GR) crop systems. This project aims to determine the degree of genetic relatedness among eight different populations of GR and glyphosate-susceptible (GS) *A. palmeri* from various geographic regions in the United States by analyzing patterns of phylogeography and diversity to ascertain whether resistance evolved independently or spread from outside to an Arizona locality (AZ-R). Shikimic acid accumulation and *EPSPS* genomic copy assays confirmed resistance or susceptibility. With a set of 1,351 single nucleotide polymorphisms (SNPs), discovered by genotyping-by-sequencing (GBS), UPGMA phylogenetic analysis, principal component analysis, Bayesian model-based clustering, and pairwise comparisons of genetic distances were conducted. A GR population from Tennessee and two GS populations from Georgia and Arizona were identified as genetically distinct while the remaining GS populations from Kansas, Arizona, and Nebraska clustered together with two GR populations from Arizona and Georgia. Within the latter group, AZ-R was most closely related to the GS populations from Kansas and Arizona followed by the GR population from Georgia. GR populations from Georgia and Tennessee were genetically distinct from each other. No isolation by distance was detected and *A. palmeri* was revealed to be a species with high genetic diversity. The data suggest the following two possible scenarios: either glyphosate resistance was introduced to the Arizona locality from the east, or resistance evolved independently in Arizona. Glyphosate resistance in the Georgia and Tennessee localities most likely evolved separately. Thus, modern farmers need to continue to diversify weed management practices and prevent seed dispersal to mitigate herbicide resistance evolution in *A. palmeri*.

## Introduction

Since the introduction of transgenic soybean, corn, and cotton in the mid-1990s, herbicide-resistant varieties of these crops have largely replaced conventional varieties in the United States (Coupe and Capel, 2016). In 1996, glyphosate-resistant (GR) (Roundup Ready) crops were commercialized and as a result global glyphosate usage rose by about 15-fold (Benbrook, 2016), dominating the current herbicide market (Duke, 2017). The widespread reliance on glyphosate to the exclusion of all other weed control methods has resulted in high selection pressure and the evolution of GR weeds, including Palmer amaranth (*Amaranthus palmeri* S. Wats.) (Culpepper et al., 2006), which is now a major threat to many U.S. food production systems (Beckie, 2011).

*Amaranthus palmeri* is a dioecious, annual species with prolific seed production, pollen-mediated gene flow due to obligate outcrossing, and high genetic variability (Franssen et al., 2001; Sellers et al., 2003; Ward et al., 2013). As a member of the *Amaranthaceae* family, *A. palmeri* is native to the southwestern United States and northwestern Mexico, having first been documented in Sonora, California, Arizona, New Mexico, and Texas in the late 19th century. During the early 20th century, the species started to spread east and northeast, probably because of human mediated seed dispersal (Sauer, 1957; Ward et al., 2013). In recent years, *A. palmeri* has expanded its distribution as far north as Ontario, Canada and as far east as Massachusetts, United States (Kartescz, 2014). The species made its first occurrence on the annual listing of most troublesome weeds in South Carolina in 1989 (Webster and Coble, 1997). By 2009 the weed was ranked the most troublesome weed in cotton in the Southern United States (Webster and Nichols, 2012; Ward et al., 2013).

Resistance to glyphosate in *A. palmeri* was first reported from a GR cotton field in Georgia in 2004. Shortly after, another case was reported from North Carolina in 2005 (Culpepper et al., 2006, 2008). As of 2017, GR *A. palmeri* was found in 27 U.S. states, Argentina, and Brazil (Scott et al., 2007; Norsworthy et al., 2008; Steckel et al., 2008; Berger et al., 2016; Heap, 2017; Küpper et al., 2017). The primary mechanism of glyphosate resistance in *A. palmeri* has been identified as the amplification of the gene encoding the target enzyme 5-enolpyruvylshikimate-3-phosphate synthase (EPSPS) which produces increased *EPSPS* transcription and protein activity (Gaines et al., 2010). The same glyphosate resistance mechanism has independently evolved in six other species (Salas et al., 2012; Jugulam et al., 2014; Lorentz et al., 2014; Chatham et al., 2015; Chen et al., 2015; Wiersma et al., 2015; Malone et al., 2016; Ngo et al., 2017). *EPSPS* gene amplification has also transferred via pollen-mediated inter-specific hybridization from *A. palmeri* to *A. spinosus* (Nandula et al., 2014).

Evolutionary models have identified that herbicide resistance dynamics are largely influenced by gene flow, seed immigration, and fitness cost (Maxwell et al., 1990). Further factors include mutation rate, the mode of inheritance, dominance of the resistance trait, seed bank turnover rate, herbicide chemistry and persistence, as well as herbicide usage patterns (Georghiou and Taylor, 1986; Jasieniuk et al., 1996; Neve, 2008). For instance, glyphosate used prior to crop emergence is predicted to have a low risk of resistance evolution while post-emergence use increases the risk, and reliance on glyphosate exclusively increases the risk even further (Neve, 2008). A simulation model for *A. palmeri* predicted that five applications of glyphosate each year with no other herbicides would result in resistance evolving in 74% of the simulated populations (Neve et al., 2011).

*Amaranthus palmeri* management is complicated by the fact that this species evolved resistance to five different modes of action (Chahal et al., 2015; Heap, 2017; Nakka et al., 2017; Schwartz-Lazaro et al., 2017), the lack of discovery of new modes of action for the past three decades, and the high cost of bringing new herbicides to the market (Duke, 2012). The overuse of and sole reliance on glyphosate and the resulting evolution of resistant weeds exhausted the lifespan of a once-in-a-century herbicide (Duke and Powles, 2008) and threatens current crop production practices by diminishing available weed management options further. Therefore, knowledge about the origin and geographical pathways of glyphosate resistance in *A. palmeri*, one of the most problematic GR weeds in the United States, is crucial to avoid repeating the same mistakes made with glyphosate with other modes of action that are still successful at controlling weeds in the field.

This study focuses on a GR population identified in a no-till cotton-wheat double crop system near Phoenix, Arizona (AZ), United States. Glyphosate was used as the sole weed management technique for the cotton portion of the production cycle for more than 10 year before glyphosate resistance was first suspected in 2012, 8 years after the first report in the species. The objective was to determine whether GR *A. palmeri* immigrated to the AZ locality from an outside location via seed or pollen-mediated gene flow, or if resistance evolved at or nearby the location in AZ independently via parallel evolution. To answer this question, single nucleotide polymorphisms (SNPs) generated by genotyping-by-sequencing (GBS) to identify numerous sequence differences at presumably random parts of the genome (Brumfield et al., 2003), were used. The GR population from AZ and seven other populations from different locations in the United States were investigated for their degree of genetic relatedness to identify patterns of phylogeography and variation on an intraspecific level.

## Materials and Methods

### Plant Material and DNA Isolation

Twelve *A. palmeri* individuals (six males and six females) from each of eight different locations in the United States were used for the analyses (**Table 1**), except for AZ-S2 for which only eleven individuals were used to leave a blank on the plate. Locations AZ-S1, AZ-S2, KS-S, GA-S and NE-S were verified as glyphosate-susceptible (GS) and locations AZ-R (Molin et al., 2017b), GA-R (Culpepper et al., 2006), and TN-R (Steckel et al., 2008) were verified as GR. The populations were collected between 2004 and 2012, except for AZ-S2 which was maintained by the USDA-ARS Germplasm Resource Information Network (accession number: Ames 5370) since its collection in 1981 and serves as an outgroup to prevent ascertainment bias (Wakeley et al., 2001; Akey et al., 2003). AZ-S1 was collected about 240 km southeast of Buckeye, AZ (AZ-R) where no agronomic crop production has occurred since the 1960s to provide a recently collected Arizona-native GS population that is fairly sympatric with AZ-R.

**Table 1:** *Amaranthus palmeri* populations used in the study, their origin and time of collection.

Abbreviation	Origin	Collection year
AZ-R	Buckeye, Arizona	2012
AZ-S1	Sahuarita, Arizona	2012
AZ-S2	Tucson, Arizona	1981
GA-R	Macon, Georgia	2006
GA-S	Worth County, Georgia	2004
KS-S	Ottawa, Kansas	2005
NE-S	Shickley, Nebraska	2011
TN-R	Jackson, Tennessee	2007


For DNA extraction, young leaf tissue was collected, immediately frozen in liquid nitrogen, and stored at -80°C. For GR samples only individuals that survived 800 g a.e. ha^-1^ glyphosate (Roundup WeatherMAX, Monsanto) were used. DNA extraction was performed following a modified cetyltrimethylammonium bromide (CTAB) extraction protocol (Doyle, 1991; Küpper et al., 2017) and quantified on a NanoDrop spectrophotometer (Thermo Scientific) followed by normalization. Gel electrophoresis and enzyme digestion with *Hind*III (Thermo Scientific) were performed on all or 10% of the samples, respectively, to confirm DNA quality and normalization.

### Herbicide Resistance Characterization

To confirm glyphosate resistance and susceptibility for the individuals used for GBS, an *in vivo* shikimate accumulation assay with excised leaf tissue (Shaner et al., 2005) was conducted. Additionally, *EPSPS* gene copy number was determined for all samples. Four-mm leaf disks from each individual were exposed to glyphosate at 0, 100, 500, and 1000 μm glyphosate for 16 h. Shikimate accumulation was measured on a spectrophotometer (Synergy 2 Multi-Mode Reader, BioTek). A shikimate standard curve was used to calculate the ng shikimate μl^-1^ accumulation above the background level. Each biological sample was run in three technical replicates for each dose.

For *EPSPS* gene copy number determination, DNA concentrations were adjusted to 5 ng μl^-1^ and primer sets (ALSF2 and ALSR2, EPSF1 and EPSR8) and qPCR conditions were used as previously described (Gaines et al., 2010). Quantitative PCR was performed using SYBR green master-mix (BioRad) on a CFX Connect^TM^ Real-Time PCR Detection System (BioRad). *EPSPS* gene copy number relative to *ALS* was determined using the 2Δ*C*_T_ method where Δ*C*_T_ = *C*_T(ALS)_ - *C*_T(EPSPS)_. Each biological sample was run in three technical replicates.

Greenhouse dose response studies were conducted to confirm pyrithiobac-sodium [acetolactate synthase (ALS inhibitor)] resistance in AZ-R with AZ-S1 as a susceptible control. The experiments took place at the University of Arizona Campus Agricultural Center in Tucson, AZ, United States. Seeds were planted in artificial soil mix in 10 cm pots and after emergence seedlings were thinned, fertilized, and irrigated as needed. ALS-inhibitor treatments included 0, 0.0001, 0.0005, 0.001, 0.002, 0.005, 0.01, 0.02, 0.05, 0.1, 0.2, 0.5, and 1 kg a.i. ha^-1^ pyrithiobac-sodium (Staple LX, DuPont) with 0.25% v/v non-ionic surfactant (Activator 90, Loveland Products). Plants were sprayed at the six-leaf stage using a CO_2_ pressurized backpack sprayer equipped with a three nozzle (TeeJet XR8001VS) boom delivering a carrier volume of 112 L ha^-1^ at 172 kPa at 4 km h^-1^. The experimental design was random with five replications per dose. Above-ground biomass was harvested 27 days after treatment (DAT), dried at 60°C and dry weight was measured.

The *ALS* gene was sequenced from three individuals each of the AZ-R, GA-R, and TN-R populations using the same DNA used for the *EPSPS* copy number test and SNP calling. *ALS* gene sequencing was conducted as previously described (Küpper et al., 2017).

### Genotyping and SNP Filtering

After DNA extraction, GBS and bi-allelic SNP calling was conducted by the Biotechnology Resource Center at Cornell University, Ithaca, NY, United States (Elshire et al., 2011). A total of 95 samples (eleven samples for AZ-S2 and twelve samples for the remaining populations) were digested with *Ape*KI, individually barcoded, run on an Illumina HiSeq2500 single-end 100 bp sequencing lane, and later trimmed to 64 bp for analysis. The GBS UNEAK pipeline in TASSEL v. 3.0.173 (Bradbury et al., 2007; Lu et al., 2013; Glaubitz et al., 2014) was used for *de novo* clustering of the sequences. The resulting SNP calls were then filtered for depth and missing values at any given locus with VCFtools v. 0.1.11 (Danecek et al., 2011) after which 4,566 filtered SNPs remained. Through further pruning, 70.4% of the filtered SNPs were excluded due to percentage of missing data points (>5%), minor allele frequency (MAF) values lower than 0.05, or more than 80% loci with more than one allele, leaving 1,351 SNPs which were informative (Supplementary Information Figure 2). Except where indicated, all analyses were performed on the panel of 1,351 SNPs.

*EPSPS* gene copies in GR *A. palmeri* individuals are randomly dispersed throughout the whole genome (Gaines et al., 2010). They can be found embedded in a complex array of repetitive elements and putative helitron sequences referred to as the ‘*EPSPS* cassette’ (Molin et al., 2017a). Because SNPs are called genome-wide, an overrepresentation of called SNPs within these sequences could potentially lead to clustering of GR individuals regardless of their actual genetic relatedness. To avoid such bias, the sequences flanking the 1,351 SNPs were aligned to the *A. palmeri* 1,044 bp *EPSPS* sequence (Gaines et al., 2010) and the 297,445 bp *A. palmeri EPSPS* cassette (Molin et al., 2017a). The 1,351 SNP sequences were also aligned to the chloroplast genome of spinach (*Spinacia oleracea*) and the mitochondrial genome of sugar beet (*Beta vulgaris*) to identify SNPs specific to the cytoplasmic regions.

### Analysis of Genetic Structure

The putative population genetic structure was explored using the model-based Bayesian analysis implemented in STRUCTURE v2.3.4 (Pritchard et al., 2000). The number of sub-populations *K* in the dataset was determined by the averaged likelihood at each *K* [In Pr (X|*K*) or In (*K*_n_)] and the variance between replicates was determined by running a continuous series of *K* = 1–15 to determine the optimal number of populations present within the 95 individuals. The analysis was carried out using a burn-in of 30,000 iterations and a run length of 100,000 Markov Chain Monte Carlo (MCMC) replications in ten independent runs. Prior knowledge about the number of populations was not included. The optimum number of clusters was predicted following the *ad hoc* statistic Δ*K* (Evanno et al., 2005) using Structure Harvester v0.6.94 (Earl, 2012). For the final *K* analysis a burn-in of 30,000 with a run length of 500,000 MCMC replications and 20 independent runs were used. To be conservative, the analyses were run assuming admixture and correlated allele frequencies (Porras-Hurtado et al., 2013). The Greedy algorithm by CLUMPP v1.1.2 (Jakobsson and Rosenberg, 2007) was used to obtain the individual and cluster membership coefficient matrices over the 20 runs which were then plotted using *distruct* 1.1 (Rosenberg, 2004).

The following information and tests were calculated in R v3.4.1. The number of alleles (*N*_a_) and allelic richness (*A*_R_) per population were calculated using the package ‘PopGenReport.’ Observed (*H*_O_) and expected heterozygosity (*H*_E_) were calculated with ‘adegenet’ (Jombart and Ahmed, 2011; Adamack and Gruber, 2014). The inbreeding coefficient (*F*_IS_) was calculated following the formula 1 - (*H*_o_/*H*_E_). Principal component analysis (PCA) was conducted using ‘SNPRelate’ and ‘gdsfmt’ (Zheng et al., 2012). Calculations for Nei’s distance (*D*_ST_) (Nei, 1972) and pairwise fixation index (*F*_ST_) among populations were performed with 1,000 bootstrap replications using ‘StAMPP’ (Pembleton et al., 2013). The analysis of molecular variance (AMOVA) (10,000 permutations) and the Mantel test (10,000 permutations) for isolation by distance analysis were performed using ‘poppr’ (Kamvar et al., 2014) and ‘adegenet’ (Dray and Dufour, 2007), respectively. The phylogenetic analysis was based on the UPGMA clustering method using the Hasegawa-Kishino-Yano (HKY) genetic distance model in the software Geneious v10.0.6.

## Results

### Herbicide Resistance Characterization

**FIGURE 1:**
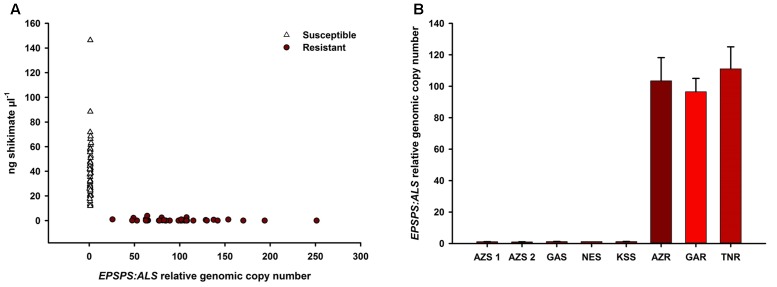
Correlation of shikimate accumulation and *EPSPS* genomic copy number in all individuals of each of the glyphosate-resistant and -susceptible *A. palmeri* populations. Shikimate accumulation was measured after incubation in 500 μm glyphosate in an *in vivo* leaf disk assay Increase in genomic copy number of *EPSPS* is relative to *ALS* as measured using qPCR on genomic DNA **(A)**. *EPSPS* genomic copy number by population **(B)**.

Glyphosate-susceptible *A. palmeri* populations showed higher shikimate accumulation (11.8–146.3 ng μl^-1^ at 500 μm glyphosate) than GR populations (0–3.8 ng μl^-1^) (**Figure 1A**) while GR populations showed higher genomic *EPSPS* copy number (individuals measured from 25- to 250-fold) than GS populations (individuals measured from onefold to twofold) (mean *EPSPS* copy number shown in **Figure 1B**). Thus, the mechanism of glyphosate resistance was determined to be *EPSPS* gene duplication in all the sampled GR populations (Gaines et al., 2010). The average copy numbers for the GR populations were within a similar range (**Figure 1B**). The 500 μm glyphosate concentration was a clear discriminating dose between GR and GS individuals.

Resistance to the ALS-inhibitors, commonly used in cotton, was suspected in AZ-R as well, thus a dose response with pyrithiobac-sodium and sequencing of the *ALS* gene was conducted. The ED_50_ values for dry weight (pyrithiobac-sodium dose causing 50% reduction in dry weight) were 6.9 and 1.3 g a.i. ha^-1^ for AZ-R and AZ-S1, respectively (*P* = 0.027) (Supplementary Information Figure 1).

Sequencing the *ALS* gene in three GR individuals each from AZ-R, GA-R and TN-R, showed that one TN and one AZ plant were heterozygous for a mutation from TGG → TTG resulting in an amino acid change from tryptophan to leucine at position 574 (W_574_L). A different AZ plant was heterozygous for a AGC → AAC mutation resulting in a change from serine to asparagine at position 653 (S_653_N). No individual carried both mutations within the same allele. The remaining individuals tested showed no mutations at these positions (Supplementary Information Table 1). Both mutations have been reported before in *A. palmeri* from Mississippi, United States, and Brazil (Molin et al., 2016; Küpper et al., 2017) while only S_653_N was reported from GA (Berger et al., 2016). The mutation at W_574_L is known to confer resistance to triazolopyrimidines, sulfonylureas, imidazolinones, and pyrimidinylthio-benzoates (including pyrithiobac-sodium), whereas the S_653_N mutation confers resistance to imidazolinones and the pyrimidinylthio-benzoates only (McNaughton et al., 2005; Whaley et al., 2006; Patzoldt and Tranel, 2007; Laplante et al., 2009; Yu et al., 2012). Both mutations are known to be inherited as a dominant trait (Tranel and Wright, 2002; Powles and Yu, 2010). It is suspected that a non-target site mechanism conferring resistance to ALS inhibitors exists (Küpper et al., 2017) and such a mechanism may also be present in AZ-R ALS-resistant individuals that lack target-site ALS mutations.

### Influence of Glyphosate Resistance Mechanism on GBS Analysis

The *EPSPS* gene was found to have five potential cutting sites for the enzyme *Ape*KI used in this GBS study, while the entire *EPSPS* cassette has 289 potential cutting sites. No SNPs were called within the *EPSPS* gene and only one SNP was called from within the *EPSPS* cassette which was removed from further analysis. Thus, the mechanism of glyphosate resistance (repetitive *EPSPS* gene copies) is not expected to influence the analysis of genetic relatedness in this case.

### Within Population Genetic Diversity

The 1,351 loci used for this study had an average percentage of missing data of 1.07% and an average MAF of 0.159. A high degree of polymorphism (MAF ≥ 0.30) was found in 14.41% of the dataset. The proportion of MAF < 0.1 was 45.37%. One AZ-S2 individual was removed from all future analysis because it was an extreme outlier. The observed number of alleles within a population ranged from 2,017 (AZ-S2) to 2,395 (KS-S), with an average of 2,217. Levels of heterogeneity were compared among populations to examine genetic variability within populations. Allelic richness (*A*_R_) ranged from 1.445 (AZ-S2) to 1.654 (KS-S) with an average of 1.560. The observed (*H*_O_) and expected heterozygosity (*H*_E_) values ranged from 0.161 (AZ-S1) to 0.219 (TN-R) and from 0.163 (AZ-S2) to 0.211 (KS-S/GA-R), respectively, with an average of 0.193. Low values for *H*_O_ indicate small effective population sizes or population bottlenecks. The *H*_O_ values in most populations were less than the *H*_E_ values (Supplementary Information Figure 3), with the exception of GA-S, TN-R and AZ-S2. The inbreeding coefficient (*F*_IS_) for these three populations was negative. AZ-R was the population with the highest *F*_IS_ value (0.121) (**Table 2**).

**Table 2:** Population information and genetic variability estimates based on 1,351 SNP loci in eight populations of *A. palmeri*.

Population	*n*	*N*_a_	*A*_R_	*H*_O_	*H*_E_	*F*_IS_
AZ-R	12	2,381	1.650	0.182	0.207	0.121
AZ-S1	12	2,299	1.581	0.161	0.181	0.110
GA-R	12	2,307	1.617	0.193	0.211	0.085
GA-S	12	2,031	1.474	0.191	0.183	-0.044
NE-S	12	2,245	1.579	0.184	0.199	0.075
KS-S	12	2,395	1.654	0.194	0.211	0.081
TN-R	12	2,059	1.477	0.219	0.183	-0.197
AZ-S2	10	2,017	1.445	0.180	0.163	-0.104


**n*, number of individuals per population; *N*_*a*_, observed number of alleles; *A*_*R*_, allelic richness; observed (*H*_*O*_) and expected (*H*_*E*_) heterozygosity; *F*_*IS*_, inbreeding coefficient.*

### Consensus Tree

**FIGURE 2:**
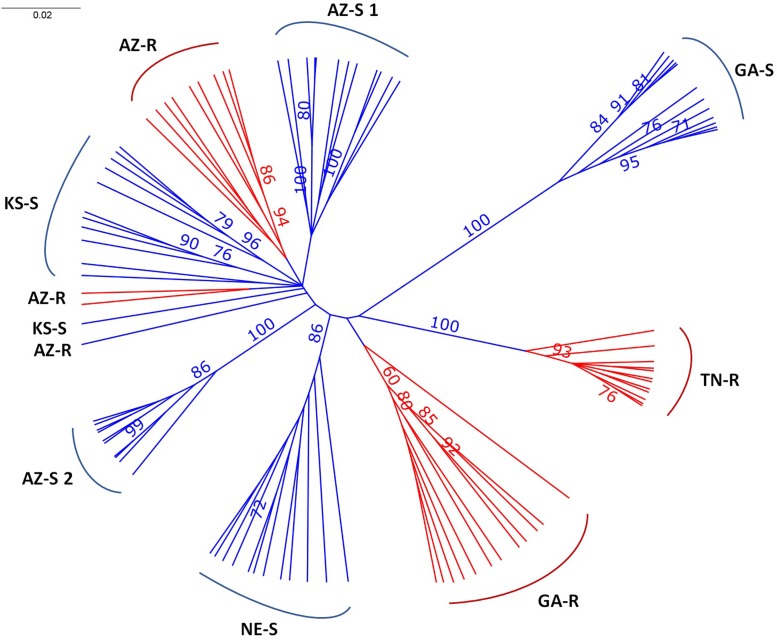
Unrooted UPGMA consensus tree after 1,000 bootstrap replications depicting the relationships of *A. palmeri* individuals from eight populations. Bootstrap values > 70% at nodes are indicated.

The consensus tree separated GA-S, TN-R, NE-S, and AZ-S2 with over 86% certainty with GAS, TN-R, and AZ-S2 being the most divergent populations. AZ-S1, AZ-R, and KS-S clustered together. Except for KS-S and AZ-R, all individuals clustered within their sampling location (**Figure 2**), The long branch lengths for the individuals indicate high within-individual genetic variability.

### Principal Component Analysis

**FIGURE 3:**
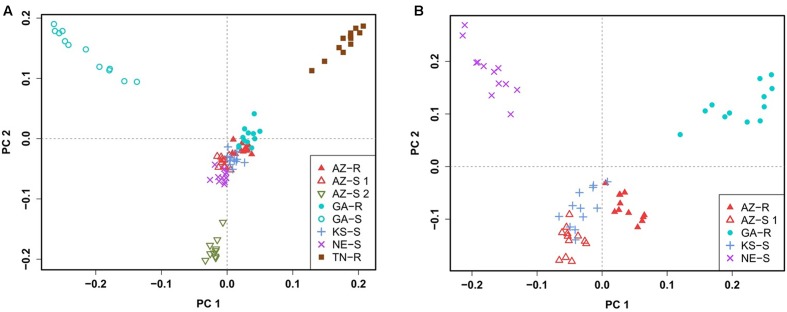
Clustering of *A. palmeri* populations based on principal component analysis (PCA) using the filtered and pruned whole dataset of 1,351 SNPs. Analysis was done on all eight populations **(A)** and on a subset of populations removing the three outlier groups AZ-S2, GA-S, and TN-R **(B)**. Each point represents an individual colored according to the collection site. Glyphosate-resistant individuals are marked by filled symbols and susceptible individuals are marked by empty symbols. Individuals from the same U.S. state have the same symbols.

To confirm this clustering, a similar pattern of differentiation among populations was constructed using PCA which is used to bring out strong patterns in the dataset based on their variance. The first two principal component (PC) axes cumulatively accounted for 16.69% of the total variation. PCA showed that all individuals clustered according to their collection site. Three distinct outgroups (GA-S, TN-R, and AZ-S2) emerged while the remaining individuals from the other five populations clustered into one group. The first dimension (PC 1) accounted for 8.91% of the variation and roughly separated GR from GS individuals (**Figure 3A**). After removing GA-S, TN-R, and AZ-S2, AZ-R did not separate from the cluster with KS-S and AZ-S1, while GA-R and NE-S clustered distinctively according to PC 2, supporting the UPGMA consensus tree. PC 1 in the second PCA on the subset of populations accounted for 6.87% of the variation in the dataset and again roughly separated GR from GS individuals. Individuals from the same population occupied different areas of the cluster, which indicates a population substructure (**Figure 3B**).

### Bayesian Analysis

**FIGURE 4:**
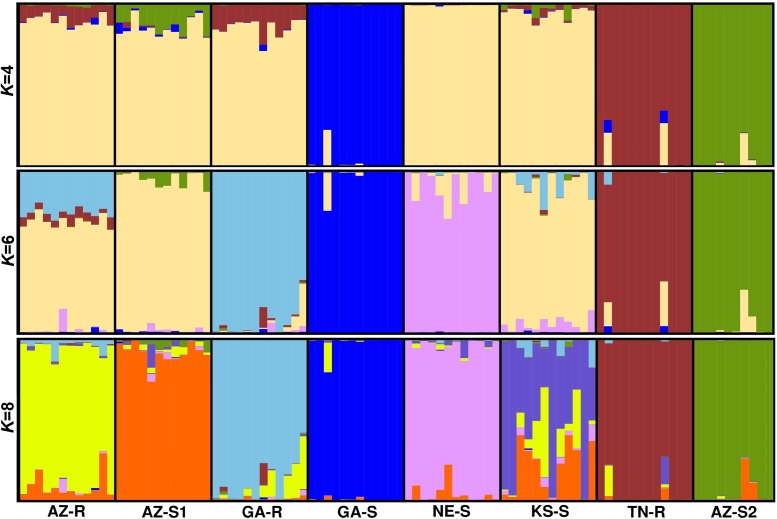
Population structure analysis with *K* = 4, *K* = 6, and *K* = 8 based on 1,351 SNPs of eight *A. palmeri* populations. Each individual is represented by a vertical bar that is divided by *K* colored segments representing the likelihood of a membership to each cluster.

Model based clustering was used to assign individuals to sub-populations based on allele frequency differences. Initially, the putative number of populations (*K*) in the dataset required to explain the total sum of genetic variation observed was determined. Evanno’s test (Evanno et al., 2005) on the whole dataset of 1,351 SNPs indicated that the *K* distribution was bimodal and that the most informative numbers of subpopulations were four and six with *K* = 6 being most probable (Supplementary Information Figure 4). At *K* = 4, consistent with the previous findings, sub-population structure analysis revealed that individuals from GA-S, TN-R, and AZ-S2 appeared distinct from the other populations. The same analysis also showed that individuals from AZ-R and GA-R shared a proportion of their alleles with TN-R while AZ-S1 shared a small proportion with AZ-S2. At *K* = 6, AZ-R, KS-S, and AZ-S1 showed the highest membership coefficient for a shared cluster (beige) while the remaining populations contained unique alleles. This is supported by a high number of shared alleles among these three populations at *K* = 8 where KS-S displayed a high degree of admixture with AZ-R and AZ-S1. Although less than with KS-S and AZ-S1, AZ-R still shared alleles with GA-R while AZ-S1 and GA-R shared none (**Figure 4**). Investigating the dataset without the three outgroups GA-S, TN-R, and AZ-S2 at *K* = 5 supports that AZ-R shares alleles with KS-S, AZ-S1, and GA-R and very few with NE-S (Supplementary Information Figure 5).

### Pairwise Comparison of Genetic Distances

As expected, very high genetic distances (*D*_ST_) (Nei, 1972) and *F*_ST_ values were found for the three outgroups (TN-R, AZ-S2, and GA-S) while the genetic distance was lower among AZ-R, AZ-S1, and KS-S. Thus, AZ-R was most closely related to AZ-S1 (*F*_ST_ = 0.052, *D*_ST_ = 0.026) and KS-S (*F*_ST_ = 0.049, *D*_ST_ = 0.028) and most distantly related to the three outgroups GA-S (*F*_ST_ = 0.201, *D*_ST_ = 0.079), TN-R (*F*_ST_ = 0.176, *D*_ST_ = 0.067), and AZ-S2 (*F*_ST_ = 0.179, *D*_ST_ = 0.067). This was further visualized by a heatmap in Supplementary Information Figure 6. The bootstrap analysis of *F*_ST_ values indicated that all populations were significantly different from each other, except for AZ-R and KS-S, where only 5% of the genetic differences between populations were attributable to their geographic origin (**Table 3**).

**Table 3:** Pairwise estimates of *F*_ST_ and Nei’s standard genetic distance (*D*_ST_) between eight *A. palmeri* populations.

	AZ-R	AZ-S1	GA-R	GA-S	NE-S	KS-S	TN-R	AZ-S2
AZ-R		0.052	0.080	0.201	0.106	0.049^†^	0.176	0.179
AZ-S1	0.026		0.126	0.202	0.108	0.051	0.215	0.170
GA-R	0.038	0.050		0.225	0.154	0.100	0.209	0.228
GA-S	0.079	0.072	0.090		0.233	0.196	0.316	0.308
NE-S	0.046	0.042	0.064	0.090		0.085	0.257	0.228
KS-S	0.028	0.026	0.046	0.077	0.039		0.191	0.171
TN-R	0.067	0.077	0.082	0.125	0.101	0.074		0.324
AZ-S2	0.067	0.058	0.088	0.115	0.084	0.064	0.122	


*Pairwise estimates of *F*_*ST*_ and *D*_*ST*_ are shown above and below the diagonal, respectively. ^†^Non-significant value (*P* > 0.05).*

### Analysis of Molecular Variance and Isolation by Distance

An AMOVA revealed that 17.78% (*P* < 0.001) of the total genetic variation was among populations, 4.87% was among individuals within a population (*P* < 0.01) and the remaining 77.35% (*P* < 0.001) of the genetic variation was within individuals (Supplementary Information Figure 7). Population differentiation exists at all levels but the variation within individuals was the largest. The high genetic variation within individuals suggests a lack of population structure, even though *F*_ST_ values up to 0.324 (**Table 3**) indicate that genetic differentiation between populations was high.

The geographical distance between any two populations ranged from about 60 to 1,930 km. The Mantel test revealed that no pattern of isolation by distance was evident between genetic and geographic distance (*R*^2^ = 0.006, *P* = 0.259). The observed correlation of 0.076 further suggests that the two distances are not associated (Supplementary Information Figure 8).

### Genetic Relatedness Based on SNPs within the Chloroplast and Mitochondrial Genome

**FIGURE 5:**
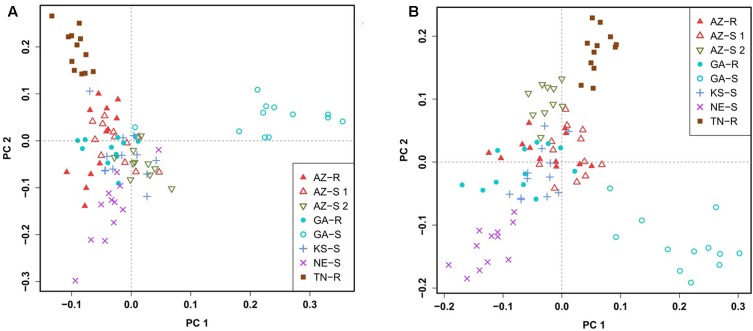
Clustering of *A. palmeri* populations based on principal component analysis (PCA) for SNPs found in all eight populations in the chloroplast genome **(A)** and the mitochondrial genome **(B)** using the filtered and pruned whole dataset. Each point represents an individual colored according to the collection site. Glyphosate-resistant individuals are marked by filled symbols and susceptible individuals are marked by empty symbols. Individuals from the same U.S. state have the same symbols.

**FIGURE 6:**
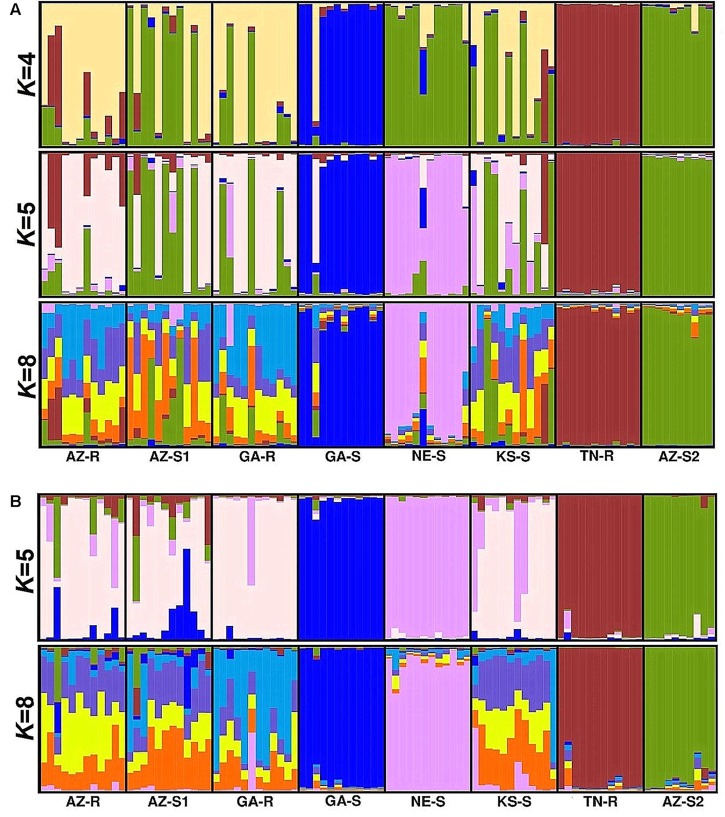
Population structure analysis with *K* = 4, *K* = 5, and *K* = 8 based on SNPs from the chloroplast genome for eight *A. palmeri* populations **(A)** and *K* = 5, and *K* = 8 based on SNPs from the mitochondrial genome for eight *A. palmeri* populations **(B)**. Each individual is represented by a vertical bar that is divided by *K* colored segments representing the likelihood of a membership to each cluster.

Forty-two SNPs specific to the chloroplast genome and fifty-four SNPs specific to the mitochondrial genome were identified. PCA with chloroplast SNPs identified GA-S and TN-R as distinct groups (**Figure 5A**). Structure analysis with the identified sub-populations of *K* = 4 and *K* = 5 (Supplementary Information Figure 9A) supported this observation. AZ-S2, however, shared considerably more alleles with NE-S, AZ-S1, KS-S, and GA-R than previously observed when including the loci from the nuclear and mitochondrial genomes (**Figure 6A**). At *K* = 8 AZ-R was closest related to AZ-S1 (*F*_ST_ = 0.058, *D*_ST_ = 0.279) (Supplementary Information Table 2).

Consistent with the analysis with all 1,351 SNPs, mitochondrial SNPs identified GA-S, TN-R, NE-S, and also AZ-S2 as distinct groups (**Figure 5B**), while the remaining populations AZ-R, AZ-S1, GA-R, and KS-S clustered together (**Figure 6B**) leaving *K* = 5 identifiable clusters among the eight populations (Supplementary Information Figure 9B). At *K* = 8, AZ-R was closest related to KS-S (*F*_ST_ = 0.053, *D*_ST_ = 0.23) (Supplementary Information Table 3).

## Discussion

Previous population genetics studies investigating the phylogeographic structure of pesticide resistant organisms reveal either a single origin (Raymond and Callaghan, 1991; Linda and Alan, 1997; Daborn et al., 2002) or, more frequently, redundant independent, parallel evolution events shaped by variations in selection pressure (Cavan et al., 1998; Anstead et al., 2005; Menchari et al., 2006; Chen et al., 2007; Pinto et al., 2007; Délye et al., 2010). As an example, it was found that glyphosate resistance in horseweed (*Conyza canadensis*) from California had multiple independent origins within the Central Valley and evolved many years before its first detection. From there it spread, possibly due to increased selection by the herbicide (Okada et al., 2013). The resistance mechanism(s) for the *C. canadensis* populations used in this study were unknown but most likely involved reduced translocation (Wang et al., 2014) and vacuolar sequestration (Ge et al., 2010). Similarly, investigations into the frequency of target site mutations in the *EPSPS* gene of GR Italian ryegrass (*Lolium perenne* L. ssp. *multiflorum*) populations (Karn and Jasieniuk, 2017) as well as simple sequence repeats (SSR) genotyping of GR common morning glory (*Ipomoea purpurea*) (Kuester et al., 2015), and GR Johnsongrass (*Sorghum halepense* L. Pers) (Fernández et al., 2013) found multiple evolutionary origins for glyphosate resistance.

Two previous studies have examined population genetics in *A. palmeri* with glyphosate resistance due to *EPSPS* gene amplification. An investigation using four genomic loci as markers of GR *A. palmeri* from several sampling sites within North Carolina suggested that adaptation to glyphosate application took place in parallel. The authors based this conclusion on the fact that four out of five identified population clusters were statistically associated with increased glyphosate resistance (Beard et al., 2014). In contrast, sequencing of selected regions of the 287 kb *EPSPS* cassette in GR populations from geographically distant locations within the United States showed strong homology between sequences and the authors concluded that the conserved nature of the cassette indicated that glyphosate resistance via amplification evolved once from a point source and then rapidly spread across the United States (Molin et al., 2017b).

Information on the factors that influence the evolutionary origin, demographic history, and geographical pathways of glyphosate resistance in *A. palmeri* is crucial for the formulation of successful strategies to delay and manage herbicide resistance. The aim of this study was to investigate population structure and genetic differentiation among eight geographically distant *A. palmeri* populations to assess if glyphosate resistance evolved in the southeastern United States and migrated to the southwestern United States, or if it evolved independently in AZ as a result of local management practices. Glyphosate resistance and susceptibility were determined by *EPSPS* copy number and shikimate assay test in all sampled individuals confirming that the resistance mechanism was *EPSPS* gene amplification. *EPSPS* genomic copy number was similar among the resistant populations; thus, spread of glyphosate resistance from a single origin is possible. GBS was used to identify numerous genome-wide sequence differences used as putative neutral markers due to its fast and simple application, cost-effectiveness and high resolution (Brumfield et al., 2003; Morin et al., 2004; Deschamps et al., 2012; Narum et al., 2013). The technique is widely applicable for studying non-model organisms, such as weeds, because the consensus of read clusters around the sequence site becomes the reference sequence and therefore a complete reference genome sequence is not required (Baxter et al., 2011; Elshire et al., 2011; Reitzel et al., 2013). For this study, 1,351 SNPs were used that remained after filtering.

Genetic diversity for each of the *A. palmeri* populations was estimated by the number of alleles, allelic richness, observed and expected heterozygosity, as well as inbreeding coefficient. The varying levels of heterozygosity found can most likely be attributed to differing collection dates and subsequent seed increase events which may have caused inbreeding depression. In particular AZ-S2, collected in 1981, is expected to have undergone severe inbreeding.

UPGMA phylogenetic tree analysis, PCA, Bayesian model-based clustering, and pairwise comparisons of genetic distances were used to determine the genetic relationship among the eight different *A. palmeri* populations and yielded congruent results. GA-S, TN-R, and AZ-S2 were genetically distinct while the remaining populations AZ-R, KS-S, AZ-S1, GA-R, and NE-S clustered together more closely. AZ-R was most closely related to KS-S, followed by AZ-S1, with GA-R being the next most similar population to AZ-R.

Cytoplasmic genomes are maternally inherited and do not undergo recombination. Thus, they permit a more conserved examination of intraspecific phylogeography in plants. They further have the potential to allow for higher differentiation (Petit et al., 2005). Chloroplast and mitochondrial SNPs were evaluated separately because they might support different phylogenies (Washburn et al., 2015; Zhu et al., 2016), since mitochondrial genomes have lower nucleotide sequence variation than chloroplast genomes (Wolfe et al., 1987). Analyses with SNPs in cytoplasmic genomes supported GA-S and TN-R to be genetically distinct. Chloroplast SNPs, however, placed AZ-S2 closer to the remaining populations than NE-S, consistent with the geographical distribution. AZ-R was closest to AZ-S1 based on chloroplast SNPs while mitochondrial SNPs placed the population closest to KS-S. Sequencing of the *ALS* gene revealed that two out of three AZ-R individuals carried a W_574_L and S_653_N mutation each, showing high diversity of the *ALS* sequence within the population. Since only the W_574_L mutation was found in one out of three individuals from TN-R, while GA-R and GA-S individuals had none (Küpper et al., 2017), mutations in the *ALS* gene do not support clustering of AZ-R and GA-R.

According to the observed population genetic structure, two scenarios are possible for AZ-R: Either glyphosate resistance evolved independently in AZ or GR *A. palmeri* from GA migrated west via KS to AZ, against the species expansion direction (Supplementary Information Figure 10). The small amount of shared sequences with GA-R and the moderate amount of shared sequences with KS-S individuals support such an introduction route, as does the chronological order of reports of glyphosate resistance (Georgia: 2004, Kansas: 2011, Arizona: 2012). AZ-R individuals shared alleles with AZ-S1 which could be attributed to crossing events with the native, GS population since resistance is likely to have been reported some time after the introduction event (Cavan et al., 1998). The high degree of unique sequence in AZ-R suggests that the exact origin of the population could not be identified. It can, however, be predicted that AZ-R individuals were not introduced from around the sampling location in TN.

Interestingly, TN-R and GA-R did not share any alleles and seemed unrelated in all analyses. Such strong population differentiation and monophyly can stem from a past divergence event and subsequent adaptation to environmental conditions through intraspecies convergent evolution (Ralph and Coop, 2015) or isolation due to limited dispersal and low connectivity (Reitzel et al., 2013). Further, agricultural practices might have strongly modified weed communities and disturbed genetic equilibrium (Menchari et al., 2007). TN and GA/NC coastal regions are geographically separated by the Appalachian mountain range and have dissimilar cropping systems with one primarily focusing on soybean and corn production and the other on cotton. As resistance to glyphosate was reported within a time frame of 2 years in these states, it is very possible that the populations represent independent glyphosate resistance origins. GA-R and GA-S, however, were genetically distinct from each other in all analyses even though collected from about 115 km apart and without any major geographical obstacles in the way. If glyphosate resistance evolved at the GA-R location, a more panmictic structure would have been expected (Chauvel and Gasquez, 1994). Such differences could be attributed to locally differing conditions, a high degree of natural spatial genetic diversity within the species, or the possibility that glyphosate resistance did not originate in GA. It is also possible that continuous selection with glyphosate created a genetic bottleneck and subsequent inbreeding of resistant individuals.

*Amaranthus palmeri* is a species with high genetic variability which makes it a challenge to draw a definite conclusion about an introduction event without very specific sampling. This study has shown that genetic relatedness does not decrease with distance. Hence, if GS individuals collected from within a 50 km radius can have the high level of genetic differentiation observed in this study (e.g., AZ-S1 and AZ-S2), it may be difficult to identify a parallel adaptation event. The nativity of *A. palmeri* to the southwestern United States and adaptation to local and heterogenous environments (Clements et al., 2004) as well as the species’ obligate outcrossing nature are drivers for heterozygosity. Genetic diversity, in turn, increases the likelihood of resistance to evolve, as does high selection pressure due to frequent usage of glyphosate which has been the case in all areas of GR *A. palmeri* collection sites. Future research should incorporate a more extensive collection of GR *A. palmeri* populations, always coupled with at least one geographically close GS population. Furthermore, all seed should be collected by the exact same sampling technique to increase the precision and accuracy with which questions of genetic relatedness and geographic migration patterns can be answered.

## Conclusion

A major management question for growers is how much of the resistance issue results from previous selection intensity from management practices in their own fields, and how much results from gene flow from neighboring fields. Although this study was not able to definitively determine whether AZ-R evolved independently or if glyphosate resistance migrated to AZ, the recent geographical expansion of *A. palmeri* to the upper United States Midwest (Kartescz, 2014), Argentina (Berger et al., 2016), and Brazil (Küpper et al., 2017) shows that migration via seed movement is an important factor for *A. palmeri*. Long-distance seed dispersal is possible through irrigation and rainfall events (Norsworthy et al., 2014), buying and selling of used harvest equipment, custom harvesting crews moving around the country (Schwartz et al., 2016), contaminated crop seed and feed, as well as transportation through migrating wildlife such as ducks and geese (Farmer et al., 2017). Aside from harvest equipment hygiene requirements, it is difficult to prevent such seed dispersal. Early detection and rapid response approaches already used in invasive species management (Westbrooks, 2004) and disease outbreaks (Fasina et al., 2014) could be useful to adopt for herbicide resistance management. Delaying resistance evolution and prolonging the utility of remaining effective modes of actions for which resistance is not yet widespread, such as synthetic auxins, glutamine synthetase-, and phytoene desaturase (PDS)-inhibitors, is critical for future *A. palmeri* management.

## Author Contributions

Conceived and designed the experiments: AK, HM, WM, and TG. Performed the experiments: AK, WM, and DG. Analyzed the data: AK and EP. Contributed materials: WM and TG. Drafted the manuscript and figures: AK. All authors contributed to the revision of the final manuscript. Any opinions, findings, conclusions, or recommendations expressed in this publication are those of the author(s) and do not necessarily reflect the view of the National Institute of Food and Agriculture (NIFA) or the United States Department of Agriculture (USDA).

## Conflict of Interest Statement

The authors declare that the research was conducted in the absence of any commercial or financial relationships that could be construed as a potential conflict of interest. The reviewer MJG and handling Editor declared their shared affiliation.
