# Supplementary material for: Population Genetic Structure in Glyphosate-Resistant and -Susceptible Palmer Amaranth (Amaranthus palmeri) Populations Using Genotyping-by-sequencing (GBS)
Source: Front Plant Sci. 2018 Jan 25;9:29. doi: 10.3389/fpls.2018.00029 (PMC5788914; doi:10.3389/fpls.2018.00029)
Supplement: Supplementary file 1 [file Data_Sheet_1.docx]

# SUPPLEMENTARY INFORMATION

# TABLES

Supplementary Information Table 1: Alignment of *ALS* amino acid sequences from a known ALS-inhibitor-susceptible *A. palmeri* (GenBank AMS38337.1) and three glyphosate-resistant individuals from Georgia (GA), Tennessee (TN), and Arizona (AZ).

| AMS38337.1-S | NNQHLGMVVQ**W**EDRFYKANRA | HQEHVLPMIP**S**GAAFKDTITE |
| --- | --- | --- |
| GA-R 1-3 | NNQHLGMVVQ**W**EDRFYKANRA | HQEHVLPMIP**S**GAAFKDTITE |
| **TN-R 1** | NNQHLGMVVQ**(W/L)**EDRFYKANRA | HQEHVLPMIP**S**GAAFKDTITE |
| TN-R 2-3 | NNQHLGMVVQ**W**EDRFYKANRA | HQEHVLPMIP**S**GAAFKDTITE |
| **AZ-R 1** | NNQHLGMVVQ**W**EDRFYKANRA | HQEHVLPMIP**(S/N)**GAAFKDTITE |
| **AZ-R 2** | NNQHLGMVVQ**(W/L)**EDRFYKANRA | HQEHVLPMIP**S**GAAFKDTITE |
| AZ-R 3 | NNQHLGMVVQ**W**EDRFYKANRA | HQEHVLPMIP**S**GAAFKDTITE |

Supplementary Information Table 2: Pairwise estimates of F_ST_ and Nei’s standard genetic distance (D_ST_) among eight *A. palmeri* populations using SNPs from the chloroplast genome. Pairwise estimates of *F*_ST_ and *D*_ST_ are shown above and below the diagonal, respectively.

|  | AZ-R | AZ-S1 | GA-R | GA-S | NE-S | KS-S | TN-R | AZ-S2 |
| --- | --- | --- | --- | --- | --- | --- | --- | --- |
| AZ-R |  | 0.058 | 0.068 | 0.223 | 0.178 | 0.215 | 0.169 | 0.205 |
| AZ-S1 | 0.028 |  | 0.096 | 0.236 | 0.167 | 0.050 | 0.252 | 0.158 |
| GA-R | 0.035 | 0.039 |  | 0.222 | 0.181 | 0.068 | 0.248 | 0.190 |
| GA-S | 0.098 | 0.088 | 0.096 |  | 0.276 | 0.187 | 0.344 | 0.236 |
| NE-S | 0.070 | 0.056 | 0.071 | 0.073 |  | 0.112 | 0.358 | 0.212 |
| KS-S | 0.035 | 0.026 | 0.036 | 0.081 | 0.046 |  | 0.215 | 0.152 |
| TN-R | 0.057 | 0.075 | 0.088 | 0.132 | 0.126 | 0.072 |  | 0.152 |
| AZ-S2 | 0.080 | 0.052 | 0.073 | 0.132 | 0.072 | 0.060 | 0.129 |  |

Supplementary Information Table 3: Pairwise estimates of F_ST_ and Nei’s standard genetic distance (D_ST_) among eight *A. palmeri* populations using SNPs from the mitochondrial genome. Pairwise estimates of *F*_ST_ and *D*_ST_ are shown above and below the diagonal, respectively.

|  | AZ-R | AZ-S1 | GA-R | GA-S | NE-S | KS-S | TN-R | AZ-S2 |
| --- | --- | --- | --- | --- | --- | --- | --- | --- |
| AZ-R |  | 0.057 | 0.091 | 0.198 | 0.152 | 0.053 | 0.213 | 0.202 |
| AZ-S1 | 0.057 |  | 0.121 | 0.143 | 0.169 | 0.047 | 0.214 | 0.191 |
| GA-R | 0.033 | 0.041 |  | 0.216 | 0.154 | 0.061 | 0.307 | 0.306 |
| GA-S | 0.070 | 0.051 | 0.076 |  | 0.230 | 0.171 | 0.338 | 0.338 |
| NE-S | 0.056 | 0.062 | 0.055 | 0.094 |  | 0.096 | 0.342 | 0.288 |
| KS-S | 0.023 | 0.022 | 0.024 | 0.062 | 0.039 |  | 0.255 | 0.228 |
| TN-R | 0.055 | 0.054 | 0.085 | 0.108 | 0.116 | 0.079 |  | 0.404 |
| AZ-S2 | 0.056 | 0.052 | 0.092 | 0.115 | 0.097 | 0.066 | 0.102 |  |

# FIGURES


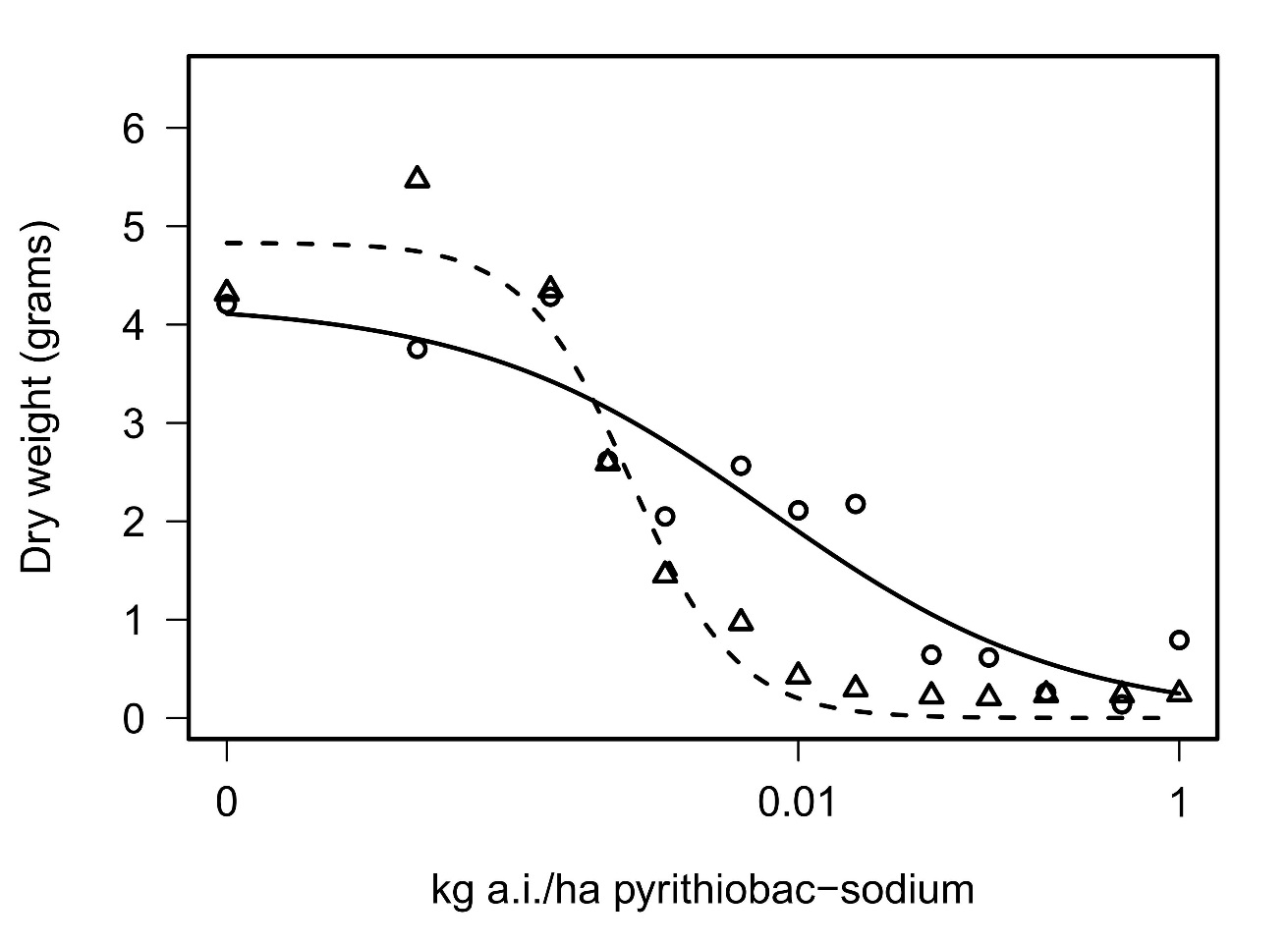


Supplementary Information Figure 1: Non-linear regression analysis of dry weight for *A. palmeri* populations AZ-R (solid line) and AZ-S1 (dashed line) 27 days after treatment (DAT) with pyrithiobac-sodium. Symbols are averages of five replicates fitted in a three-parameter log-logistic model.


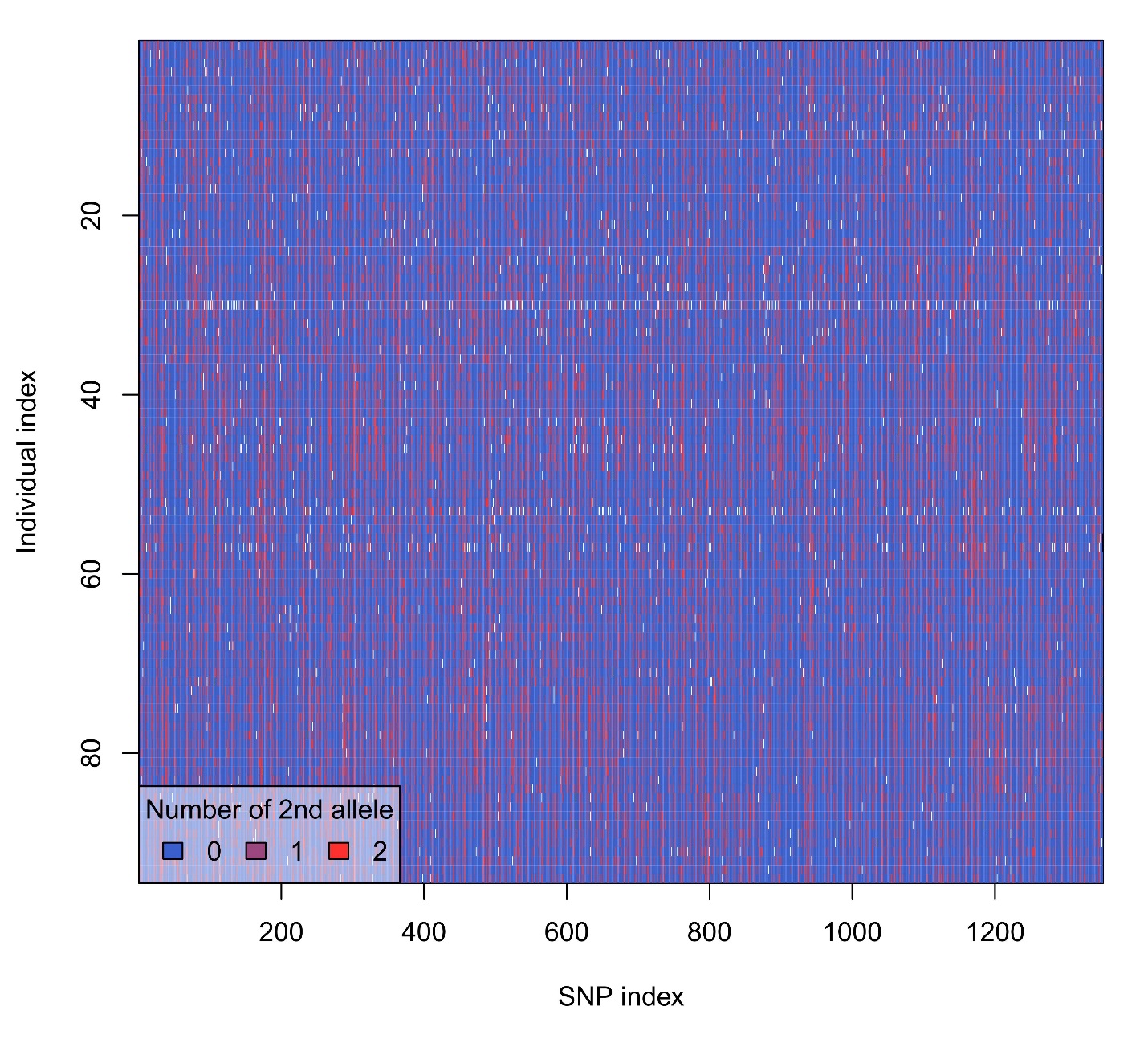


Supplementary Information Figure 2: Allele structure of individuals versus loci. Blue/0 = homozygous for the most common allele (major allele), violet/1 = heterozygous, red/2 = homozygous for the least common allele (minor allele), white = missing data.


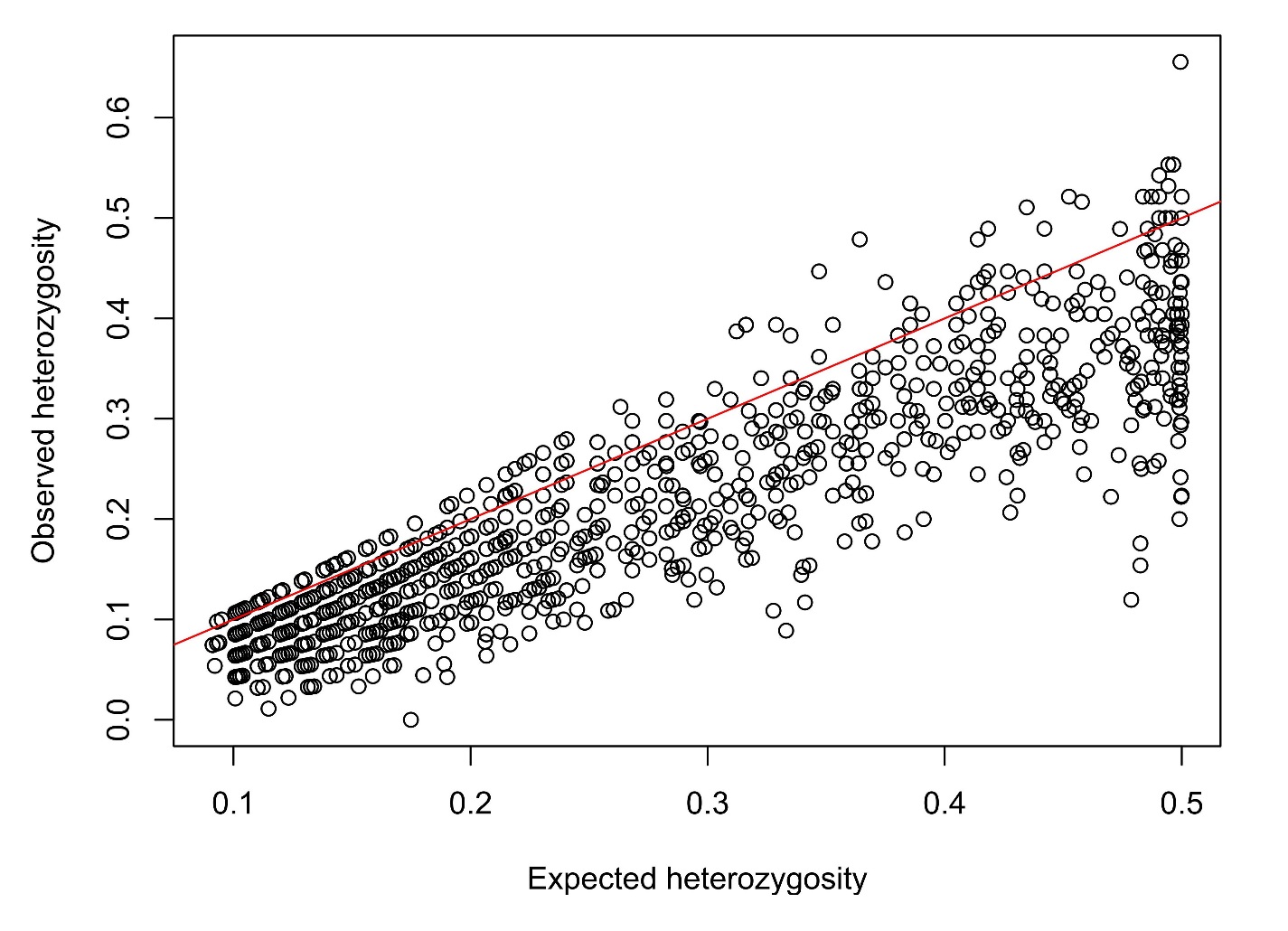


Supplementary Information Figure 3: Observed heterozygosity as a function of expected heterozygosity per locus for the whole dataset of 1,351 SNPs.


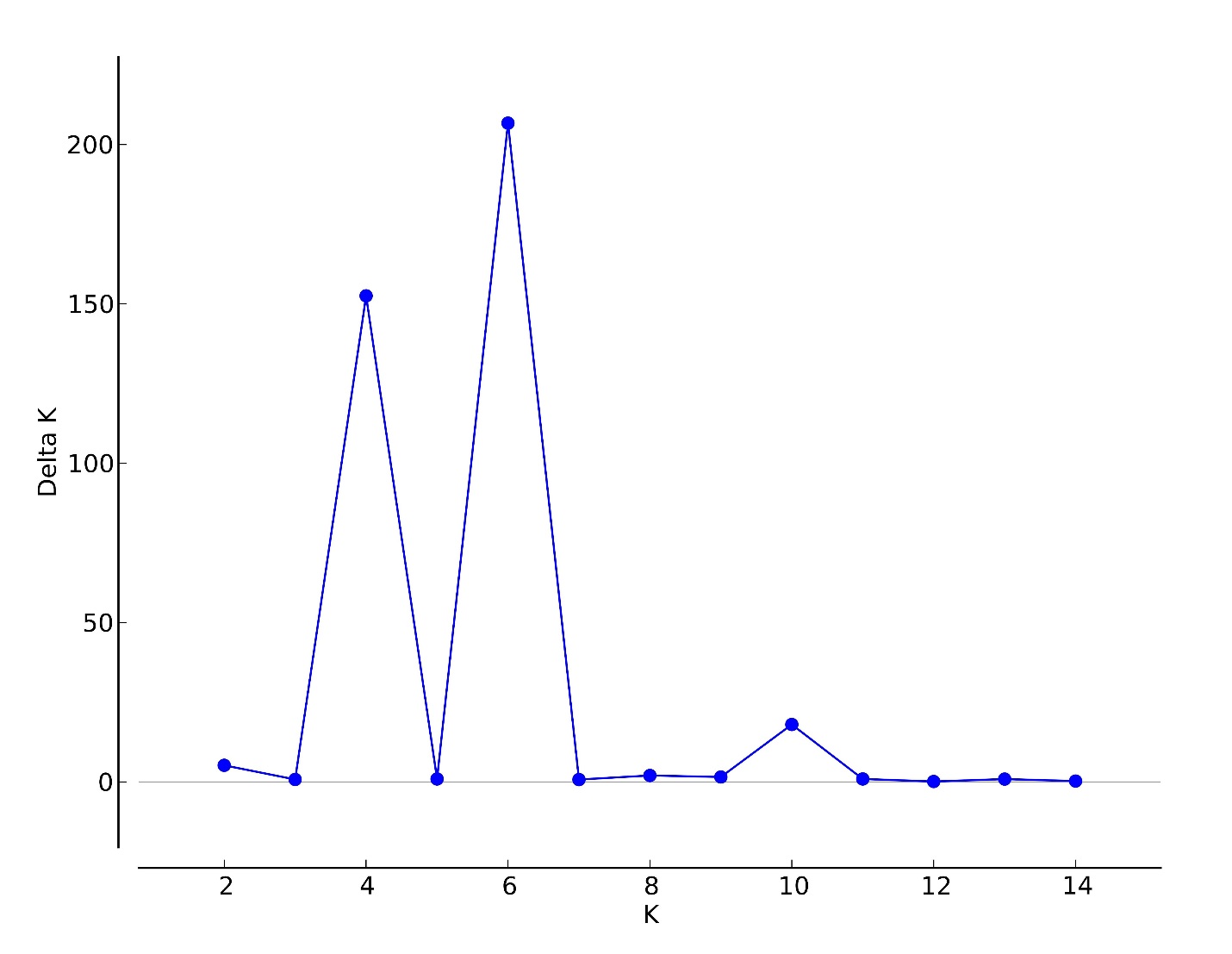


Supplementary Information Figure 4: *Ad hoc* ΔK test (Evanno et al., 2005) with the whole dataset of 1,351 SNPs. The analysis suggests *K* = 4 and *K* = 6 to be the most likely amount of sub-populations.


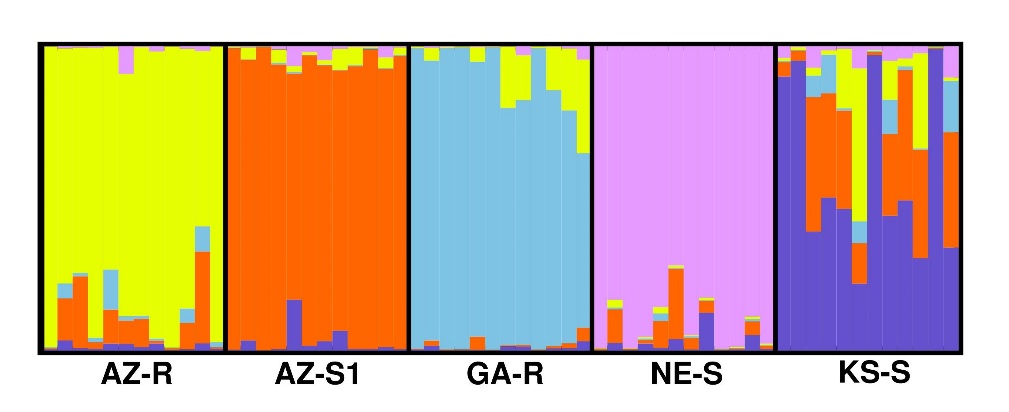


Supplementary Information Figure 5: Population structure analysis with *K* = 5 for the 1,351 SNPs of the five *A. palmeri* populations which clustered together in Fig. 3A, excluding GA-S, TN-R, and AZ-S2. Each individual is represented by a vertical bar that is divided by *K* colored segments representing the likelihood of a membership to each cluster.


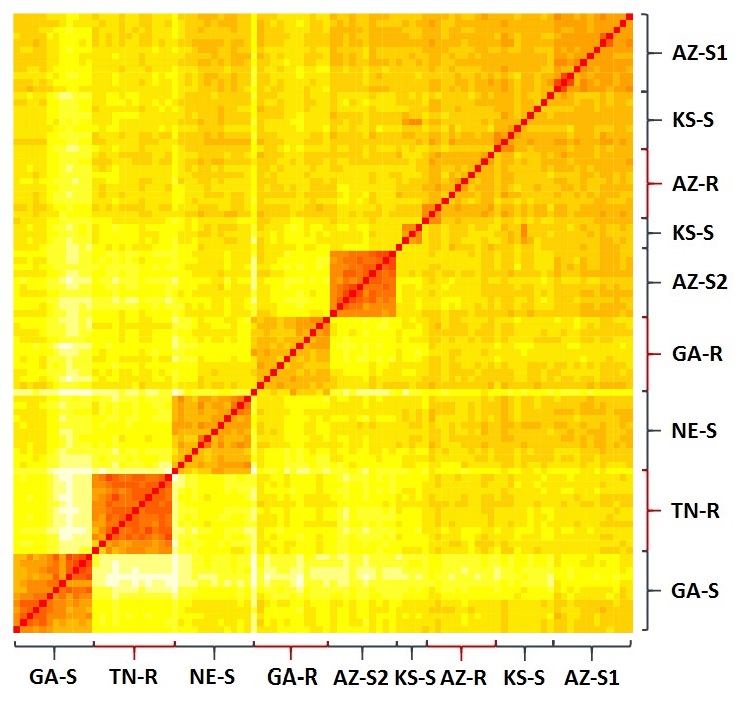


Supplementary Information Figure 6: Visualization of Nei’s distance (1972) as a heatmap. Each pixel refers to one individual being compared to another individual. Red marks a low degree of genetic distance while white and light yellow mark a high degree of genetic distance between these individuals. The diagonal line shows an individual being compared to itself while red squares indicate members of the same population. The missing square structures for KS-S, AZ-S1, and AZ-R are an indication for the high genetic variability with the population.


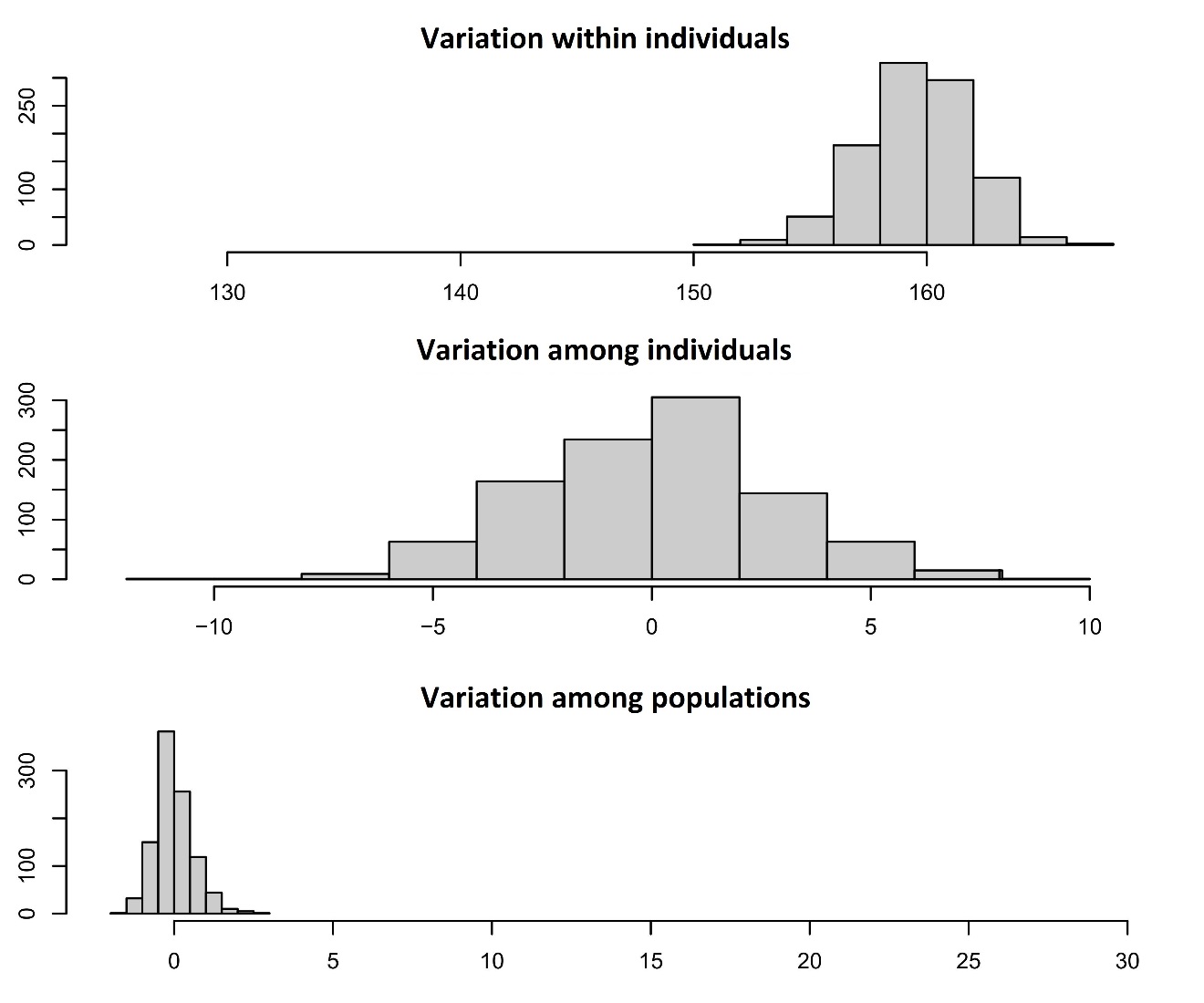


Supplementary Information Figure 7: Three histograms that represent the distribution of randomized strata for variation within individuals, variation among individuals and variation among populations. Variation within individuals is very high while variation among individuals and among populations is low.


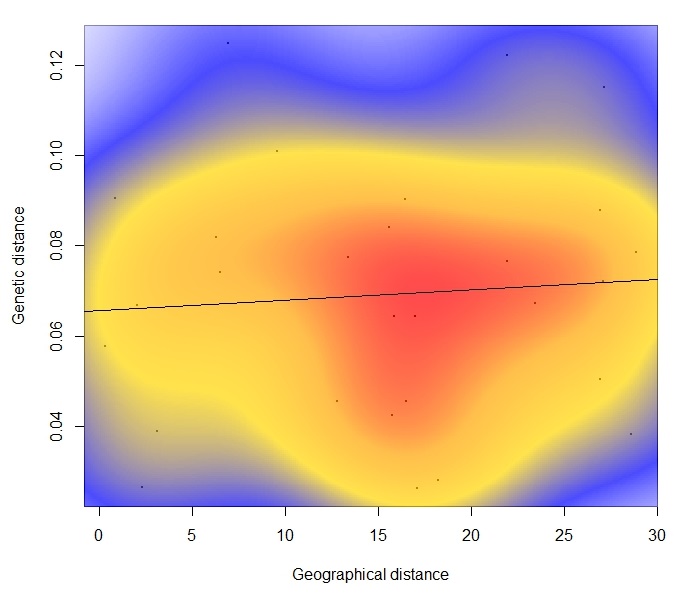


Supplementary Information Figure 8: Correlation of genetic and geographical distance of eight *A. palmeri* populations from several U.S. states. The denser areas in the plot indicate sub-groups. With an R^2^ of 0.006, no correlation between genetic and geographical distance was found.


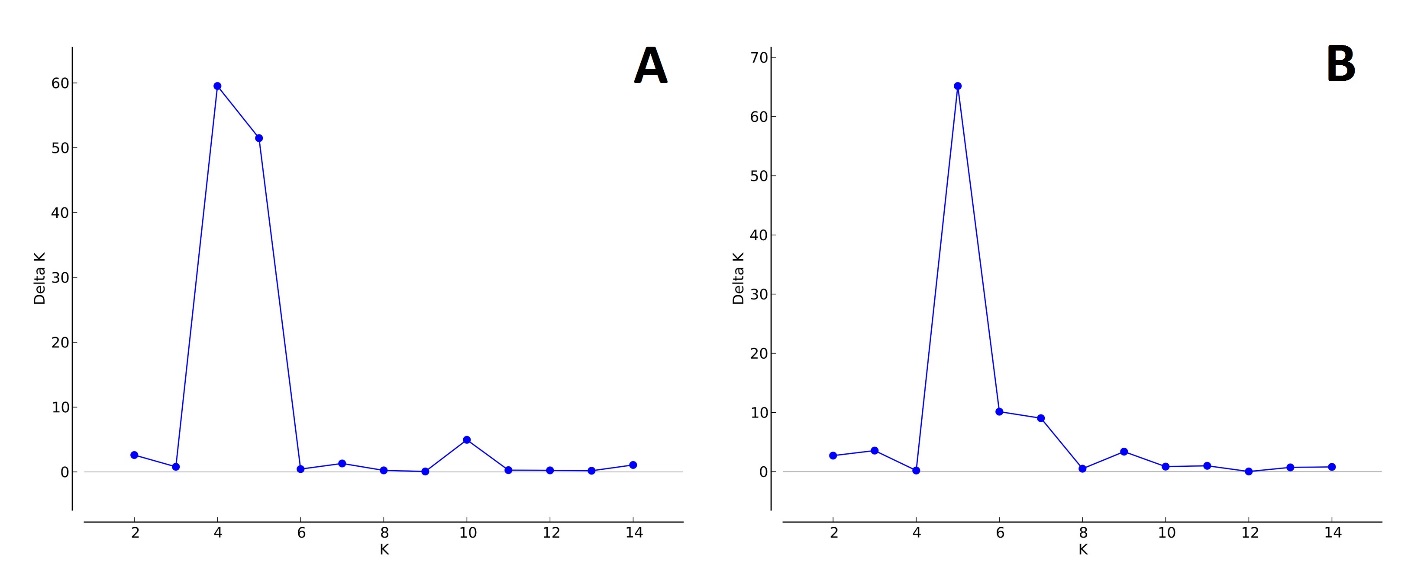


Supplementary Information Figure 9: *Ad hoc* ΔK test with SNPs from the chloroplast genome (A) and the mitochondrial genome (B). The analysis suggests *K* = 4 and *K* = 5 as well as *K* = 5 to be the most likely amount of sub-populations, respectively.


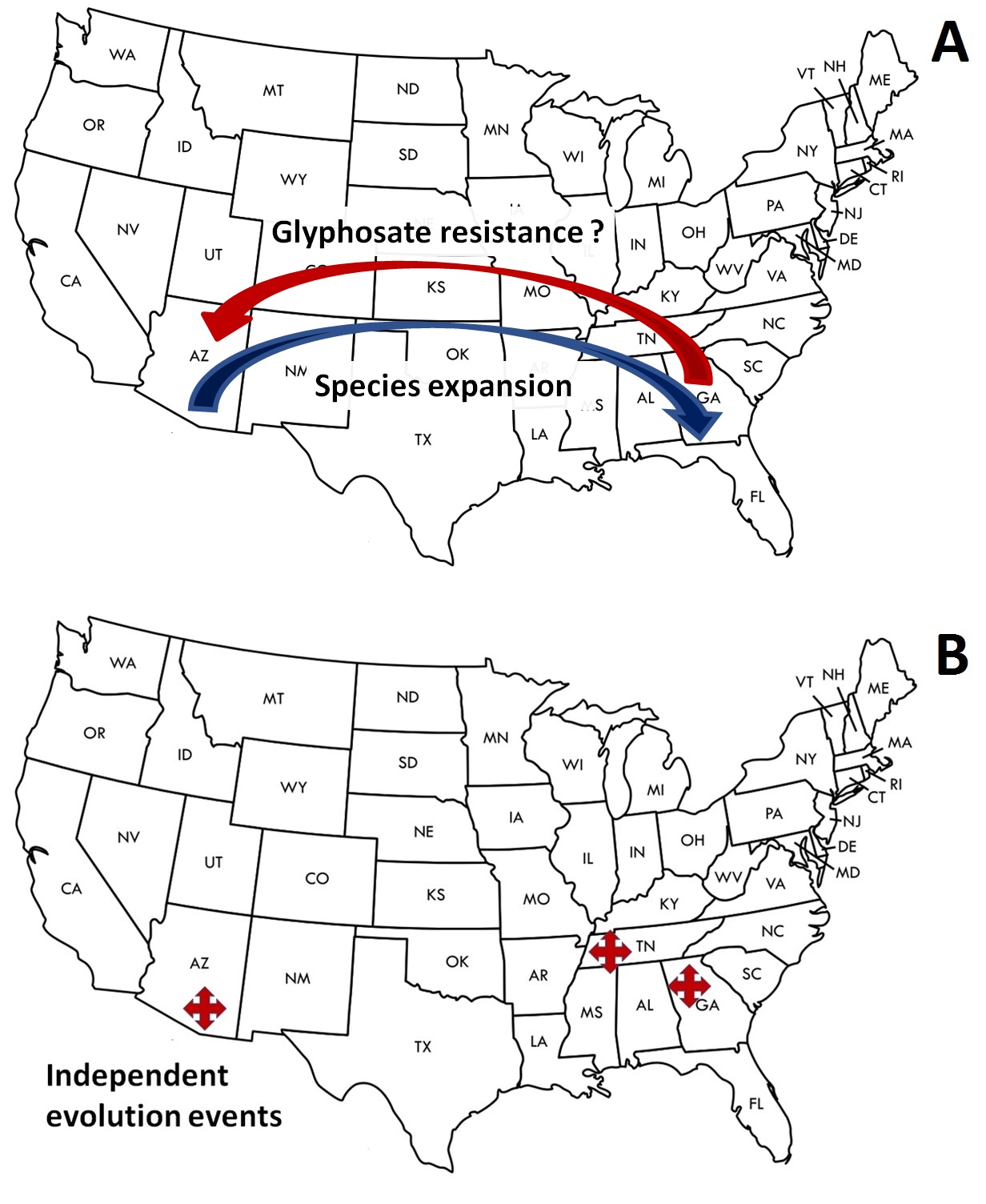


Supplementary Information Figure 10: Two possible scenarios of glyphosate resistance origin in *A. palmeri* with either glyphosate resistance spread from Georgia over Kansas to Arizona in the counter-direction of species expansion to the southeast (A) or independent evolution events in Arizona, Georgia and Tennessee (B).
